# Structural determinants of CO_2_-sensitivity in the β connexin family suggested by evolutionary analysis

**DOI:** 10.1038/s42003-019-0576-2

**Published:** 2019-09-04

**Authors:** Valentin-Mihai Dospinescu, Sarbjit Nijjar, Fokion Spanos, Jonathan Cook, Elizabeth de Wolf, Maria Assunta Biscotti, Marco Gerdol, Nicholas Dale

**Affiliations:** 1 School of Life Sciences, University of Warwick, Coventry, CV4 7AL UK; 2 Dipartimento di Scienze della Vita e dell’Ambiente, Università Politecnica delle Marche, 60131 Ancona, Italy; 3 Dipartimento di Scienze della Vita, Università di Trieste, 34127 Trieste, Italy

**Keywords:** Molecular evolution, Physiology

## Abstract

A subclade of connexins comprising Cx26, Cx30, and Cx32 are directly sensitive to CO_2_. CO_2_ binds to a carbamylation motif present in these connexins and causes their hemichannels to open. Cx26 may contribute to CO_2_-dependent regulation of breathing in mammals. Here, we show that the carbamylation motif occurs in a wide range of non-mammalian vertebrates and was likely present in the ancestor of all gnathostomes. While the carbamylation motif is essential for connexin CO_2_-sensitivity, it is not sufficient. In Cx26 of amphibia and lungfish, an extended C-terminal tail prevents CO_2_-evoked hemichannel opening despite the presence of the motif. Although Cx32 has a long C-terminal tail, Cx32 hemichannels open to CO_2_ because the tail is conformationally restricted by the presence of proline residues. The loss of the C-terminal tail of Cx26 in amniotes was an evolutionary innovation that created a connexin hemichannel with CO_2_-sensing properties suitable for the regulation of breathing.

## Abstract (web summary)

Valentin-Mihai Dospinescu et al. present an evolutionary analysis of the connexin Cx26, by comparing the structure and function of this protein in fish, *Xenopus* and reptiles. They find that Cx26 acts as a CO_2_ sensor in mammals and reptiles, but not fish or amphibia, and loss of its C-terminal tail is linked to this gain of function.

## Introduction

There are 20 connexin genes in the human genome^1^. This large number of variants in the connexin gene family, implies diversity of cellular and physiological function which may depend on the precise properties of the different connexins. Connexins form gap junctions, which comprise two hexameric hemichannels in the membranes of adjacent cells docked together to form a dodecameric complex. Gap junctions are aqueous pores that permit ion flow and transfer of small molecules between the coupled cells. In addition to this canonical function of coupling cells, the hexameric hemichannels can have an independent function by acting as large conductance plasma membrane channels^2^. Hemichannels are a particularly important mechanism for the release of ATP into the extracellular space^3–5^. We have discovered that the β connexins, Cx26, Cx30 and Cx32 are modulated by CO_2_^6^. Hemichannels of each of these connexins can be opened by CO_2_. In the case of Cx26, this direct CO_2_-gated hemichannel opening, and subsequent release of ATP, mediates an important part of respiratory chemosensitivity^7^. There are other important molecules that may also contribute to respiratory chemosensitivity — these include pH-sensitive channels and receptors such as the TASK channels^8,9^ and GPR4^10,11^. The physiological significance of the CO_2_ sensitivity of Cx30 and Cx32 has not yet been elucidated.

We have analyzed the structural basis of the CO_2_ dependent modulation of Cx26 hemichannels in detail, and have discovered that it most likely depends upon the carbamylation of Lys125, and formation of a salt bridge from the carbamylated lysine to Arg104 of the neighbouring subunit (a “carbamate bridge”)^12^. This carbamate bridge increases the time that the hemichannel spends in the open configuration. Our structural studies have allowed us to define a “carbamylation motif” that is present in CO_2_-sensitive connexins, but absent from those that are insensitive to CO_2_^12^.

Very recently we have discovered that there are two actions of CO_2_ mediated via the carbamylation motif. Whereas CO_2_ *opens* hexameric hemichannels, it has the opposite effect and causes *closure* of the gap junctions^13^. Mutational analysis shows that this closing effect of CO_2_ on the Cx26 gap junction is most likely mediated by its binding to the same residues that effect hemichannel opening. Both effects of CO_2_ are likely therefore to be mediated via the carbamylation motif.

Several authors have examined the evolution of the β connexin family^1,14^. In this paper we use our insights about the nature of the carbamylation motif to further refine our understanding of the phylogenetic occurrence of this motif, and hence CO_2_-sensitivity, in the β connexin family. This approach has given us new insight into the structural determinants of the CO_2_ sensitivity of both gap junctions and hemichannels and has shown that the carbamylation motif was present in the ancestor of all gnathostomes. Interestingly, it is a common feature of the amniote species tested that their Cx26 hemichannels lack an extended C-terminal tail and are consequently sensitive to CO_2_. This suggests that the common ancestor of all extant amniotes had already evolved CO_2_-sensitive Cx26 hemichannels.

## Results

### Molecular phylogenetic and microsyntenic analysis

**Fig. 1:**
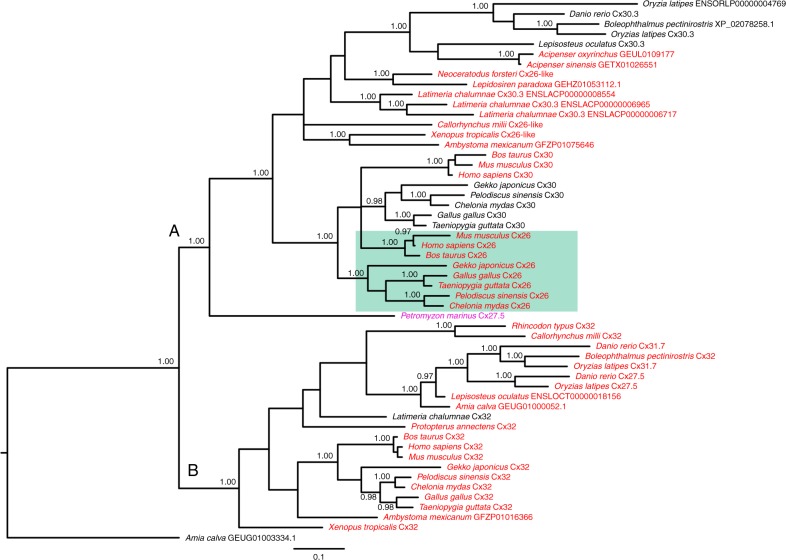
Molecular phylogenetic tree of *Cx26*, *Cx30* and *Cx32* genes. Bayesian Inference on amino acid sequences belonging to the main vertebrate lineages. Numbers close to nodes indicate posterior probability values (>0.95). *Amia Calva* GEUG01003334.1 (Cx31-like) was used as the outgroup. If the species name is red, the sequence possesses the carbamylation motif. The green box indicates *Cx26* with a short C-terminal tail. The *Cx27.5* sequence for lamprey is in purple as it has elements of the carbamylation motif but is very different from any other motif (see text for discussion). Two main clades are apparent and labelled A and B

**Fig. 2:**
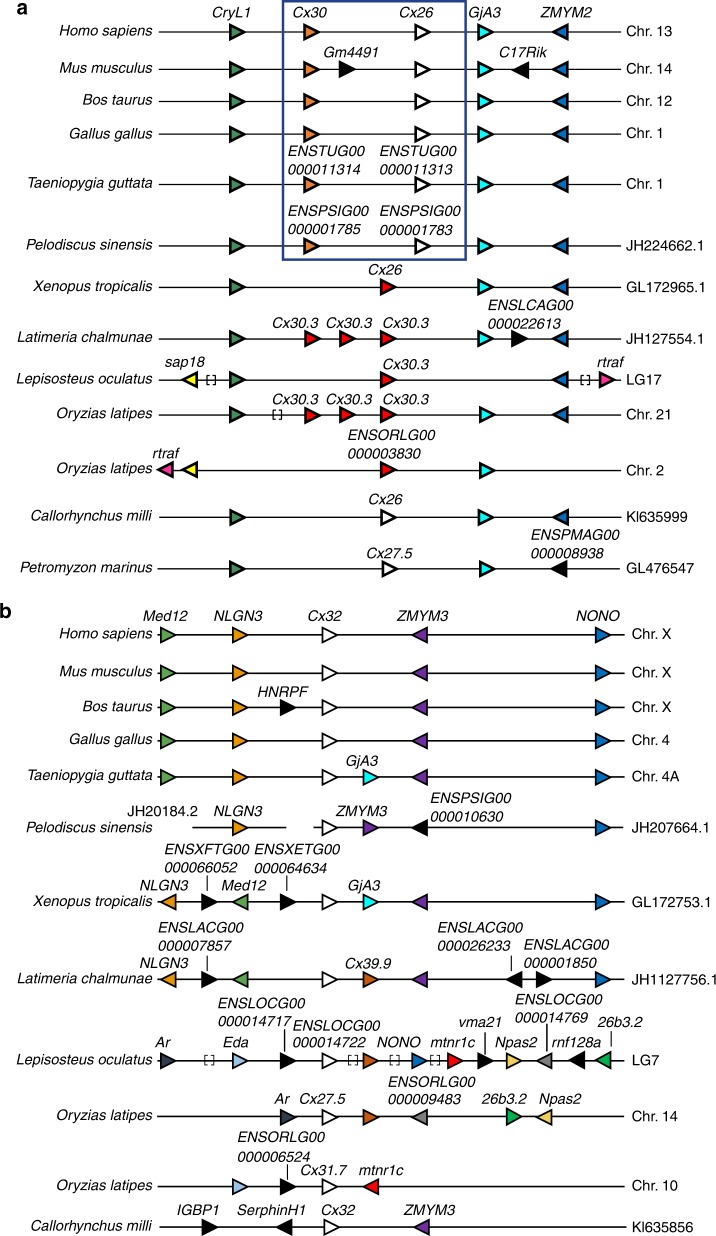
Microsynteny of the genomic regions harboring *Cx26*, *Cx30* and *Cx32* genes in the main vertebrate lineages. **a** Comparative analysis of microsynteny related to the genomic region harboring *Cx26* and *Cx30* genes in the main vertebrate lineages. **b** Comparative analysis of microsynteny related to the genomic regions harboring *Cx32* in the main vertebrate lineages. Arrow-heads indicate gene direction and colour help to follow orthology relations. Black arrow-heads indicate genes not showing evident orthology relations. Lines underneath genes indicate syntenic arrangement. Square brackets indicate the presence of multiple genes in that genomic region. The blue box indicates the *Cx26* and *Cx30* genes present in amniotes

The amino acid sequences from fifty-three β connexin family members, from 24 vertebrate species, were used for molecular phylogenetic analysis (Fig. 1 and Supplementary Table 1). Additional species that we inspected (and support our conclusions) but did not include in the phylogenetic analysis of Fig. 1 are listed in Supplementary Table 2, and shown in Supplementary Fig. 1. The resulting tree topology exhibited two main clades named A and B, supported by high values of posterior probability. Clade A consisted of the sequence Cx27.5 of the jawless *Petromyzon marinus* and a cluster comprising the Cx26, Cx30 and Cx30.3 sequences of gnathostomes. These sequences are distributed further into two subgroups: one containing the sequences for Cx26 and Cx30 of reptiles, birds, and mammals; the other containing sequences belonging to elephant shark, actinopterygians, coelacanth, lungfish, and amphibians. The analysis was not able to establish the correct orthology and paralogy relations in the clade A. The microsyntenic analysis of the chromosome region harbouring these genes showed a conserved pattern of flanking genes from lamprey to mammals (Fig. 2a). This indicates that the common craniate ancestor of agnathans and gnathostomes already had this genomic arrangement. Furthermore, this analysis reveals that in amniotes two genes are located between *CryL1* and *GjA3*, corresponding to *Cx30* and *Cx26*, respectively (Blue box, Fig. 2a). This result, together with the phylogenetic analysis, supports the hypothesis proposed by Abascal and Zardoya^14^ that these connexin genes are derived from a duplication event that occurred in the common amniote ancestor of reptiles, birds, and mammals. However, for the clade containing *Cx30* and *Cx26* sequences of amniotes, our phylogenetic analysis does not allow the orthology and paralogy to be ascertained probably because mechanisms such as gene conversion, common in tandemly arranged genes, may have hidden the real relationships. In the amphibian *Xenopus tropicalis,* only one gene is located between *CryL1* and *GjA3*. This gene is annotated as *Cx26* but is probably orthologous to the ancestral gene from which *Cx26* and *Cx30* of amniotes originated. The orthology relation documented for *X. tropicalis* can be extended to all sequences of non-amniote organisms here analyzed. The presence of more genes in this chromosome region, as for example in coelacanth, is due to lineage-specific duplication. Thus, the sequences of amphibians and lungfish are most probably more closely related evolutionarily to the ancestral gene from which the amniote *Cx26* and *Cx30* genes arose and are more correctly named *Cx26-like*. For simplicity, we shall refer to these genes as *Cx26*.

The clade B included the sequences corresponding to Cx32 of the cartilaginous and actinopterygian fish and of sarcopterygians. Furthermore, the analysis allowed elucidation of the orthology relation between these sequences and those of actinopterygians. This finding is in agreement with the microsyntenic analysis performed between the main vertebrate lineages (Fig. 2b). Indeed, the flanking regions of *Cx32* gene shared several genes indicating a common origin. However, the pattern between birds and mammals is more conserved compared to that of actinopterygians probably due to genomic rearrangements. The genes named *Cx27.5* and *Cx31.7* in teleosts are ohnolog genes thus derived from the lineage-specific genomic duplication event that affected the genome of these organisms^15,16^.

The carbamylation motif is present in both clades A and B. In clade B, this motif is almost universally present. Interestingly in clade A, there are two substantial branches where it has been lost: non-mammalian, amniote *Cx30*; and actinopterygian *Cx30.3*. Interestingly, Cx30.3 may play a similar role in cochlea of fish to that of Cx26 of mammals^17^. The lamprey has a version of the carbamylation motif that is unusual: it possesses Arg104 and Lys125, but there are two prolines in the sequence (Pro124, Pro123). No sequence from any other vertebrate that we have studied possesses this sequence and, given the steric restrictions that the two proline residues would introduce, it is questionable whether Lys125 could be properly oriented to form a salt bridge to Arg104 following carbamylation. The carbamylation motif therefore is definitely present in the ancestor to all gnathostomes, and may have evolved in the agnathans too, albeit in a heavily modified form. Over the two clades, almost all sequences are characterized by a long C-terminal tail. The only notable exception to this is Cx26 of amniotes (green box, Fig. 1, see also Supplementary Table 2, Supplementary Fig. 1) in which the C-terminal tail has been truncated to only a few amino acids.

### Sensitivity of Cx26 hemichannels to CO_2_

**Fig. 3:**
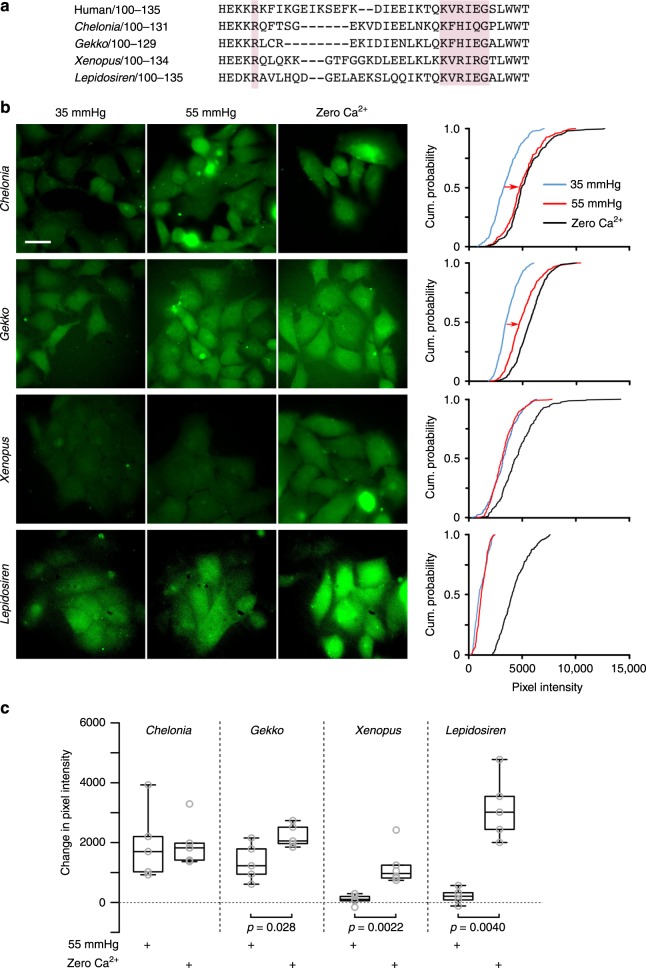
Amniote Cx26 hemichannels can be opened by CO_2_. **a** Amniotes, amphibia and lungfish share the CO_2_ carbamylation motif (highlighted boxes). **b** Images of dye loading in response to CO_2_, and the zero Ca^2+^ positive control. **c** Summary data showing that reptile Cx26 hemichannels open to CO_2_ but those of *Xenopus* and *Lepidosiren* do not (expressed as the change in pixel intensity from control 35 mmHg PCO_2_). Mann Whitney test used in comparisons. Box and whisker plots: box, first and third quartiles; horizontal line, median; whiskers, the furthest point that lies no more than 1.5 times the interquartile range from the median. Each point is the median change in pixel intensity from an independent transfection. Scale bar 20 µm

**Fig. 4:**
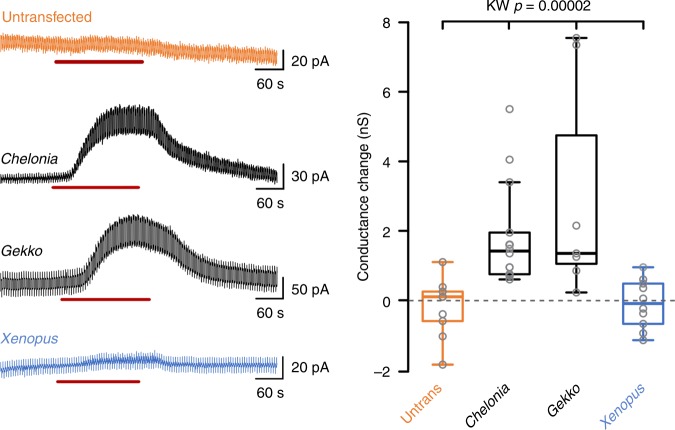
Whole-cell patch clamp recordings demonstrate a CO_2_-sensitive conductance in HeLa cells expressing *Chelonia* and *Gekko* Cx26, but not in untransfected parental HeLa cells or HeLa cells expressing *Xenopus* Cx26. Red bar indicates application of 55 mmHg hypercapnic saline. Cells clamped at −50 mV, with steps to −40 mV to assess whole-cell conductance. KW, Kruskal–Wallis ANOVA. Box and whisker plots: box, first and third quartiles; horizontal line, median; whiskers, the furthest point that lies no more than 1.5 times the interquartile range from the median. Each point represents an independent patch clamp recording (replicate)

Our sequence comparisons show that the carbamylation motif is present in sarcopterygian fish and tetrapods. As we have already established the CO_2_-sensitivity of Cx26 hemichannels coded by the mammalian and avian genes^6,18^, we tested whether the Cx26 hemichannels of reptiles (*Chelonia* and *Gekko*), amphibia (*Xenopus*) and lungfish (*Lepidosiren*), also exhibit CO_2_-dependent opening. To evaluate this, we used our well established and validated dye-loading assay^6,12,18–21^ to test whether we could detect entry of carboxyfluorescein into HeLa cells expressing these Cx26 genes during a CO_2_ challenge (Fig. 3). As a positive control to check for functional expression of hemichannels in the membrane we used a zero Ca^2+^ stimulus which is effective at opening hemichannels by a CO_2_-independent mechanism, and provides a measure of maximal dye loading to compare to the CO_2_-dependent dye loading. All four tested Cx26 genes possessed a carbamylation motif, very similar to that of human Cx26 (Fig. 3a). However only HeLa cells expressing the reptilian Cx26 exhibited CO_2_-dependent dye loading (Fig. 3b, c). Nevertheless HeLa cells expressing all four genes showed dye loading to the zero Ca^2+^ stimulus demonstrating the presence of functional hemichannels (Fig. 3b, c). We confirmed these results by means of whole-cell patch clamp recordings to demonstrate the presence of a CO_2_-dependent conductance in HeLa cells expressing *Chelonia* and *Gekko* Cx26, but not in HeLa cells expressing *Xenopus* Cx26 or non-transfected HeLa cells (Fig. 4). Thus, the Cx26 hemichannels from *Xenopus* and *Lepidosiren* are not sensitive to CO_2_. Within the species tested therefore, only Cx26 hemichannels from amniotes possess CO_2_ sensitivity.

### The C-terminal tail controls CO_2_-sensitivity of Cx26

**Fig. 5:**
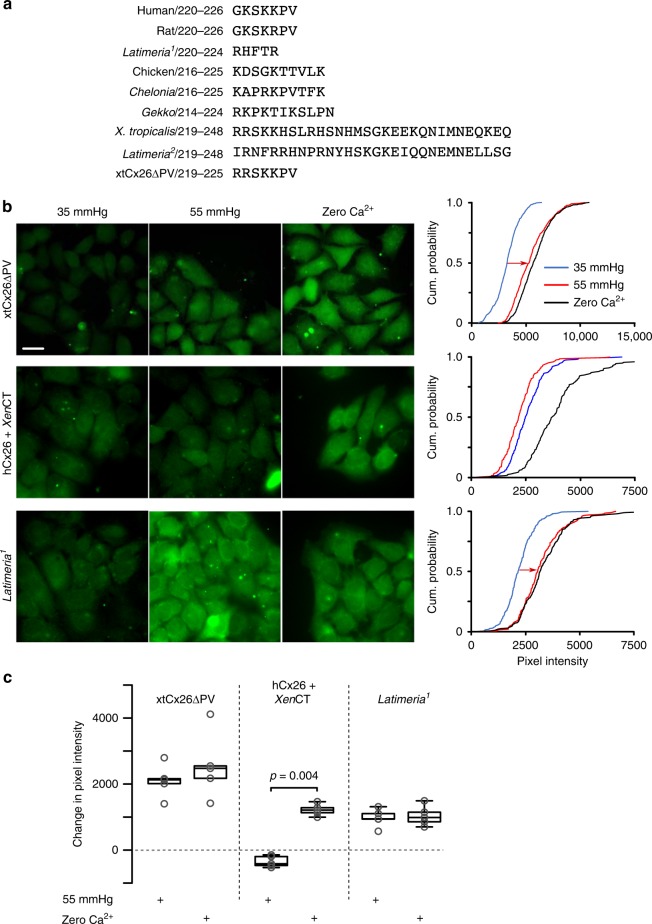
The extended C-terminal tail of non-amniote sarcopterygians inhibits the CO_2_ sensitivity of Cx26 hemichannels. **a** Comparison of the C-terminal tail of Cx26 in amniote and non-amniote vertebrates. **b** Removal of the C-terminal tail of Xenopus and substitution of PV for final two residues (xtCx26ΔPV) gives good expression of the modified Cx26 and demonstrates gain of CO_2_ sensitivity. Addition of the *Xenopus* C-terminal tail to human Cx26 (hCx26 + *Xen*CT) causes loss of CO_2_ sensitivity. In *Latimeria*, a copy of the Cx26 gene lacking the C-terminal tail is CO_2_ sensitive. 1. XP_014348762.1, 2. ENSLACG00000007568. **c** Summary data showing the increase in dye loading with the 55 mmHg PCO_2_ and zero Ca^2+^ stimuli (expressed as the change in pixel intensity from control 35 mmHg PCO_2_). Box and whisker plots: box, first and third quartiles; horizontal line, median; whiskers, the furthest point that lies no more than 1.5 times the interquartile range from the median. Each point represents the median change in pixel intensity from the control saline (35 mmHg PCO_2_) from an independent transfection. Scale bar 20 µm

**Fig. 6:**
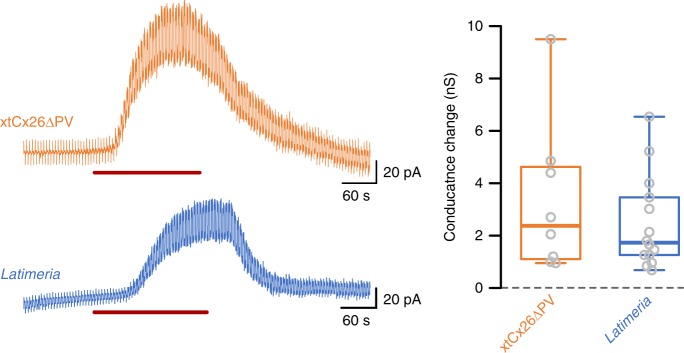
Whole-cell patch clamp recordings demonstrate a CO_2_-sensitive conductance in HeLa cells expressing xtCx26ΔPV and *Latimeria* Cx26. Red bar indicates application of 55 mmHg hypercapnic saline. Box and whisker plots: box, first and third quartiles; horizontal line, median; whiskers, the furthest point that lies no more than 1.5 times the interquartile range from the median. Each point represents an independent patch clamp recording (replicate)

On inspection of amphibian, coelacanth (Fig. 5a) and lungfish Cx26 amino acid sequences, we noticed that Cx26 of these species possessed a C-terminal tail considerably longer than that of the Cx26 of amniotes. We therefore tested whether removal of this extended C-terminal tail could restore the CO_2_ sensitivity of Cx26 in these species. We truncated the tail of *Xenopus* Cx26 and altered the final two residues so they were the same as in mammalian Cx26 to improve trafficking (xtCx26ΔPV). HeLa cells expressing this truncated Cx26 now demonstrated both CO_2_-dependent dye loading (Fig. 5b, c) and CO_2_-dependent conductance changes (Fig. 6). Conversely the addition of the *Xenopus* C-terminal tail to human Cx26 (hCx26 + *Xen*CT) effectively abolished the CO_2_ sensitivity of human Cx26 hemichannels (Fig. 5b, c). Finally, evolution has performed the same manipulation for us: *Latimeria* (Coelacanth) has 3 different homologues of Cx26, two of which have a long C-terminal tail, and in a third this C-terminal tail has been truncated to the same length as the human gene (Fig. 5a). We therefore tested whether the truncated *Latimeria* Cx26 gene encodes CO_2_-sensitive hemichannels. We found that HeLa cells expressing this truncated gene did indeed exhibit CO_2_-dependent dye loading (Fig. 5b, c) and CO_2_-dependent whole-cell conductance changes (Fig. 6). We therefore conclude that the two critical criteria necessary for CO_2_ sensitivity in Cx26 hemichannels are the lack of an extended C-terminal tail and the presence of the carbamylation motif. This condition is met by the Cx26 of many amniote species.

### CO_2_-sensitivity of Cx32 hemichannels

**Fig. 7:**
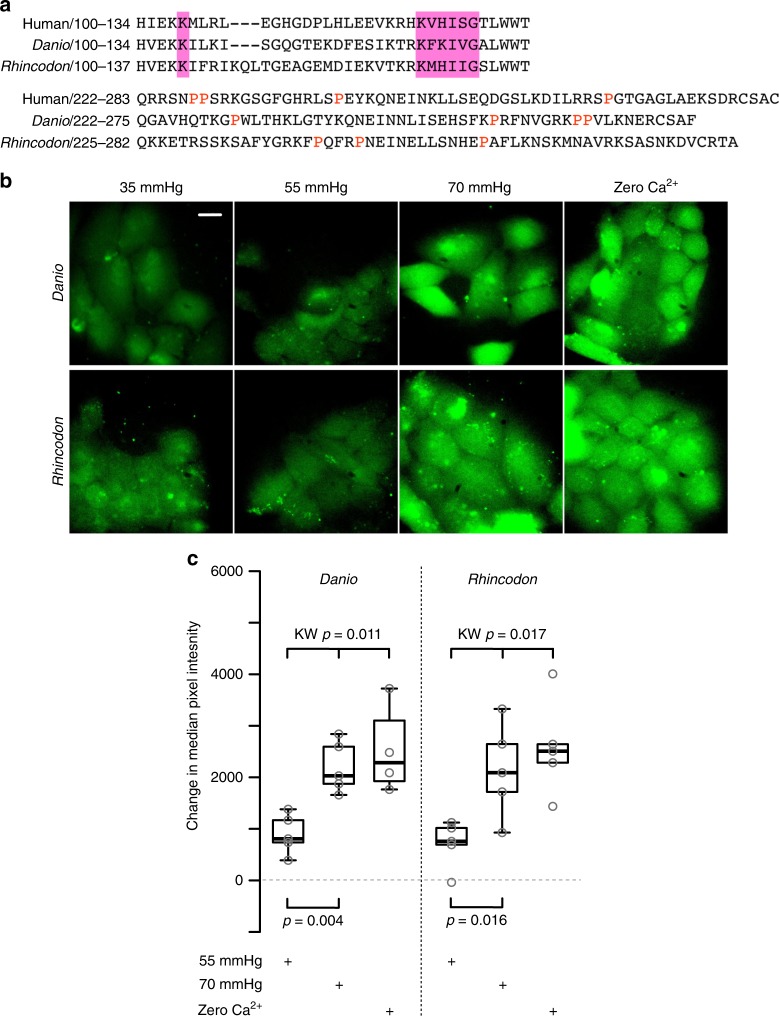
Cx32 hemichannels in fish can be opened by high levels of CO_2_. **a** Sequence of the carbamylation motif (pink box) and extended C-terminal tail in Cx32 of human, *Danio* and *Rhincodon*. Note the presence of proline residues in the extended C-terminal tails of each species. **b** Dye loading experiments showing a small amount of dye loading with 55 mmHg CO_2_, and substantial loading with 70 mmHg PCO_2_ and zero Ca^2+^. **c** Box and whisker plots: box, first and third quartiles; horizontal line, median; whiskers, the furthest point that lies no more than 1.5 times the interquartile range from the median. All points the median change in pixel intensity from the 35 mmHg control saline. Each point represents result from an independent transfection. Scale bar 20 µm

We have previously shown the Cx32 hemichannels from rat can be opened by CO_2_, but require higher levels of PCO_2_ than Cx26^6^. Inspection of the *Cx32* amino acid sequence in a variety of actinopterygian and cartilaginous fish revealed the presence of a carbamylation motif very similar to that of human *Cx32* (Fig. 7). This implies that this motif was already present in the common ancestor of Chondrichthyes and Osteichthyes. Unlike *Cx32*, *Cx26* in actinopterygian fish does not have the carbamylation motif except in a very few cases for primitive fish (Fig. 1, Supplementary Table 2, Supplementary Fig. 1). Furthermore, *Cx32* (like *Cx30*, which is also CO_2_-sensitive) possesses a long C-terminal tail, which in *Cx26* would abrogate CO_2_ sensitivity.

We therefore tested whether *Danio* (Zebrafish) and *Rhincodon* (whale shark) Cx32 hemichannels were CO_2_ sensitive (Fig. 7). We found that there was a small amount of CO_2_ dependent dye loading at a PCO_2_ of 55 mmHg, and robust dye loading at a PCO_2_ of 70 mmHg (Fig. 7b, c). Like human Cx32 hemichannels, the fish homologues are sensitive to CO_2_ but require a substantially higher stimulus than those of amniote Cx26 to open them^6^.

**Fig. 8:**
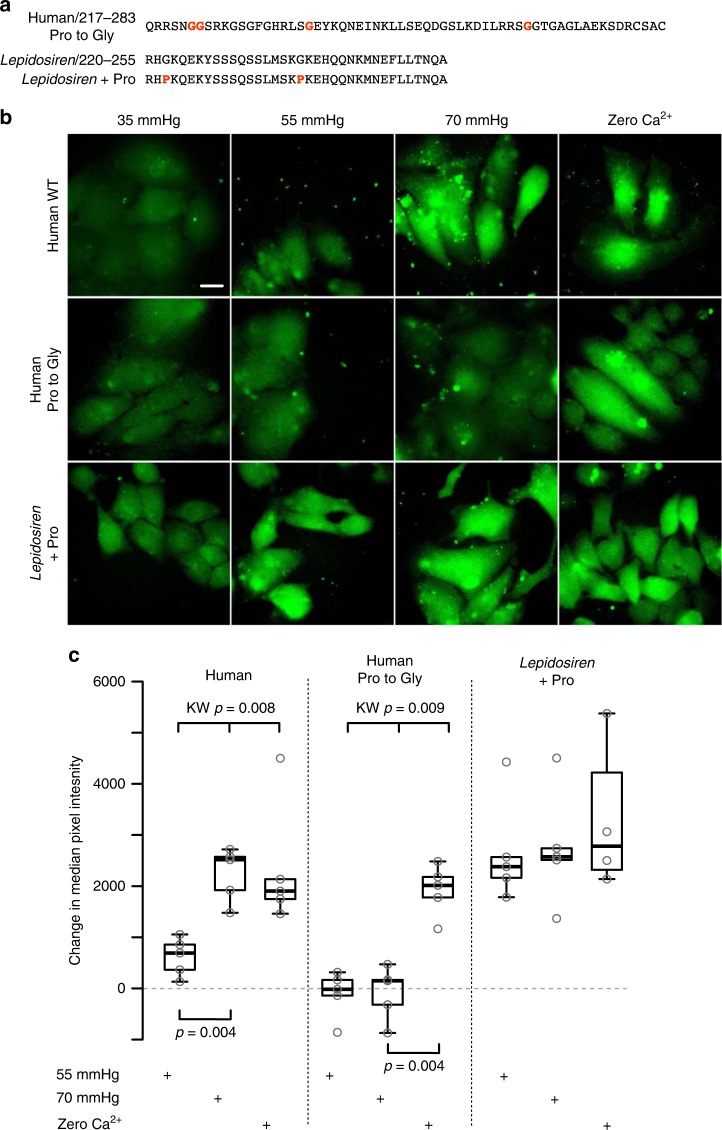
Prolines in the extended C-terminal tail, permit CO_2_ sensitive opening of hemichannels. **a** Sequences of: modified human Cx32 C-terminal tail with glycine in place of proline; and *Lepidosiren* Cx26 C-terminal tail, showing the glycines that were changed to proline. **b** Human Cx32 hemichannels can be opened by CO_2_. Mutation of prolines in the extended C-terminal tail abolishes CO_2_ sensitivity (Human Pro to Gly). Introduction of two prolines into the *Lepidosiren* C-terminal tail gives a gain of CO_2_ sensitivity (compare to Fig. 3b, c). **c** Summary data showing the change in median pixel intensity compared to the control (PCO_2_ 35 mmHg) from five independent replications for each connexin. KW, Kruskal–Wallis ANOVA, pairwise comparisons Mann Whitney. Box and whisker plots: box, first and third quartiles; horizontal line, median; whiskers, the furthest point that lies no more than 1.5 times the interquartile range from the median. Each point represents result from an independent transfection. Scale bar 20 µm

This leads us to the intriguing question of why the extended C-terminal tail in Cx32 does not abrogate the CO_2_ sensitivity of hemichannels, whereas in Cx26 it does. By inspecting the sequences of *Cx32* we noticed that, unlike the amphibian and lungfish *Cx26*, there were multiple proline residues in the C-terminal tail (Fig. 8a). As prolines will conformationally restrict an unstructured peptide sequence, we hypothesized that the resulting structure could prevent the C-terminal tail of Cx32 interfering with the CO_2_-dependent opening of the hemichannels. We therefore mutated all proline residues to glycine (Fig. 8) in the C-terminal tail of human Cx32. This completely removed the sensitivity of Cx32 hemichannels to CO_2_ (Fig. 8b, c). We also performed the converse experiment: Cx26 hemichannels of *Lepidosiren* are not sensitive to CO_2_. To explore whether introduction of prolines into the C-terminal tail gave a gain of CO_2_-sensitivity in *Lepidosiren* Cx26, we changed two glycine residues in the extended C-terminal tail to proline (Fig. 8a). Remarkably, the presence of the prolines in the C-terminal tail conferred CO_2_ sensitive opening on *Lepidosiren* Cx26 hemichannels (Fig. 8b, c). We conclude that hemichannels of Cx32, and by extension Cx30, both of which have extended C-terminal tails, are CO_2_ sensitive because the presence of proline residues prevents the extended tail from interfering with either CO_2_ binding to the carbamylation motif or the subsequent conformational change that leads to hemichannel opening.

### The ancestral function of the carbamylation motif

The carbamylation motif exists in *Cx32*, including *Cx32* of shark, suggesting a very ancient evolutionary origin to at least the ancestor of all gnathostomes. The motif has also been conserved in homologues of *Cx26* (amphibian and lungfish) in which the hemichannels are not CO_2_ sensitive. It is notable that only a single base change is needed to effect the K125R mutation, which destroys CO_2_ sensitivity^12^. That the motif has been preserved over 400 MY, suggests selection pressure to maintain an important biological function. This in turn suggests that the original function of the motif in Cx26 must have been something other than opening the hemichannel.

We have recently discovered that modestly elevated CO_2_ has two actions on mammalian Cx26: (1) opening of hemichannels; and (2) to closing of gap junctions^13^. As mutations that remove the ability of the hemichannel to open to CO_2_ also remove the ability of CO_2_ to close Cx26 gap junctions, the CO_2_ dependent closure of Cx26 gap junctions seems to depend on CO_2_ binding to the same residues that open the hemichannel i.e. the carbamylation motif. To explore whether gap junction closure might be the ancestral function of the carbamylation motif in Cx26, we examined whether CO_2_ could close *Lepidosiren* Cx26 gap junctions.

**Fig. 9:**
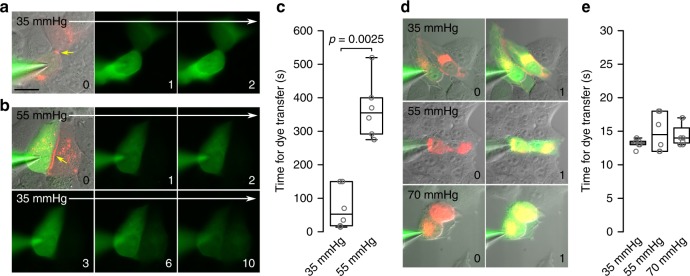
The ancestral function of the carbamylation motif in Cx26 but not Cx32 is to close gap junctions. **a** Images showing rapid permeation of NBDG (within 1 min of establishing whole-cell recording) through the *Lepidosiren* Cx26 gap junction when PCO_2_ is 35 mmHg. In the images, red shows the distribution of the mCherry-tagged *Lepidosiren* Cx26, green is NBDG fluorescence, the yellow arrow indicates the gap junction between the cells. The numbers in bottom right hand corner are minutes after establishing whole-cell recording configuration. Scale bar, 20 µm. **b** Permeation of NBDG through the gap junction is delayed by elevated PCO_2_. The cells were perfused with hypercapnic saline (PCO_2_ 55 mmHg) for 2 min following breakthrough, and then transferred to control saline (PCO_2_ 35 mmHg). Significant permeation of the dye into the coupled cell is apparent only by 6th minute. **c** Summary data showing the effect of PCO_2_ on delaying permeation of dye through the gap junction to the coupled cell. **d** Cx32 Gap junctions are insensitive to CO_2_. NBDG permeates rapidly (within seconds after establishing the whole-cell configuration) through the gap junction at all levels of PCO_2_. **e** Summary data showing that there is no difference in the time required for dye transfer between coupled cells at different levels of PCO_2_. *N* = 6 for each treatment (independent replicates); box and whisker plots show the median and interquartile range (IQR), with the whisker indicating the furthest point that lies no more than 1.5 times the IQR from the median. The time for dye transfer was calculated to be when the acceptor cell had reached 10% of the fluorescence of the donor cell

We tested whether exposure to different levels of PCO_2_ could affect the movement of a fluorescent tracer from a single cell (loaded via a patch pipette) through gap junctions to coupled cells. A PCO_2_ stimulus of 55 mmHg prevented dye-spread through *Lepidosiren* gap junctions, and permeation of the dye only occurred once the saline had been changed to a PCO_2_ of 35 mmHg (Fig. 9a–c). This demonstrates that *Lepidosiren* Cx26 gap junctions are closed by CO_2_ even though the hemichannels are insensitive (Fig. 3b, c). The extended C-tail therefore does not interfere with binding of CO_2_ to the carbamylation motif or the conformational changes that this induces in the gap junction to close it. Presumably the C-terminal tail prevents conformational change leading to hemichannel opening. By association we reasoned that the gap junctions of Cx32 may also be sensitive to CO_2_. To our surprise, we found that permeation of fluorescent tracer occurred through the gap junction very rapidly at all levels of PCO_2_ tested (Fig. 9d, e). Thus, gap junctions of Cx32, unlike those of Cx26, are not sensitive to CO_2_ at these doses.

## Discussion

By studying CO_2_ sensitivity in the β connexin clade in a number of different phylogenetic groups from shark to mammals, we have unexpectedly revealed new structural requirements that determine the actions of CO_2_ on these connexins. As we have already described, the carbamylation motif is a necessary requirement for CO_2_-dependent modulation^12^. The presence of this motif engenders CO_2_-dependent closure of gap junctions of Cx26. For Cx26 hemichannels, a further structural condition is required to gain CO_2_-dependent opening: the truncation of the C-terminal tail. When this tail is present, it prevents this opening action of CO_2_. That the extended tail does not prevent gap junction closure, strongly suggests that the tail does not interfere with the carbamylation event, or indeed the conformational change leading to gap junction closure, but instead prevents the conformational changes required to open the hemichannels once CO_2_ has bound. A parsimonious explanation might be that the extended C-terminal tail stabilises the closed conformation of both the gap junction and hemichannel when CO_2_ is bound.

However, our analysis highlights a further essential structural feature of the C-terminal tail for CO_2_-dependent hemichannel opening. The very long tail of Cx32 still permits CO_2_-dependent opening of Cx32 hemichannels, albeit at significantly higher levels of PCO_2_. The presence of prolines in this tail permits hemichannel opening in response to CO_2_. Changing these prolines to glycine abrogates the CO_2_ sensitivity, and introducing prolines into the C-terminal tail of the non-sensitive *Lepidosiren* Cx26 gives a gain of function and permits CO_2_-dependent opening of the hemichannel. Presumably, the proline residues introduce a degree of conformational restriction into the C-terminal tail that prevents the extended tail from interfering with hemichannel opening.

Given that there are two functions of the carbamylation motif -gap junction closing and hemichannel opening, what was the original ancestral function of this motif? Our finding that some Cx26 orthologues (*Xenopus*, lungfish) possess the motif, but do not open to increased CO_2_, strongly suggests that modulation of gap junction activity might be the original function. This receives further support from our demonstration that the lungfish Cx26 gap junctions can indeed be closed by CO_2_.

The level of PCO_2_ tested in this study (55 mmHg) is high compared to the typical levels of PCO_2_ found in lungfish and amphibia. This dose of PCO_2_ is near saturating for Cx26, which in mammals and birds is sensitive to changes of PCO_2_ over the range 20–60 mmHg^6,18^. Ventilation in *Lepidosiren* responds to changes in PCO_2_ over the range 21–42 mmHg and is controlled by central chemoreceptors that are sensitive to both pH and PCO_2_^22^. Breathing in *Rana catesbeiana* responds to changes in PCO_2_ from 6–42 mmHg^23^. While the hemichannels of both these species are insensitive to CO_2_, the *Lepidosiren* gap junctions were completely closed by a PCO_2_ of 55 mmHg. It is therefore possible that the CO_2_-sensitivity of Cx26 gap junctions (i.e. involving partial closure) at lower levels of PCO_2_ could contribute to the chemosensory control of ventilation in these species. Further experimental data is needed to test this proposition.

Gap junctions of Cx32 are insensitive to the levels of PCO_2_ used in this study. This suggests that in Cx32 the original function of the carbamylation motif was to open hemichannels. Cx32 hemichannels of fish and humans can be opened by sufficiently high levels of PCO_2_ (55–70 mmHg). In entirely water breathing vertebrates, such as elasmobranch or actinopterygian fish, systemic PCO_2_ is only slightly above the ambient^24^. It is therefore very unlikely that systemic PCO_2_ would reach a range of 55–70 mmHg sufficient to open Cx32 hemichannels. Thus, Cx32 hemichannels in fish are most probably not used as systemic CO_2_ sensors to regulate breathing in the way that Cx26 hemichannels are used in mammals. The possible functions of the CO_2_-sensitivity of Cx32 remain enigmatic. The preservation of the carbamylation motif in Cx32 over a long evolutionary period, suggests that there is indeed some important physiological function for the CO_2_-sensitivity of this connexin. A possible hypothesis is that the Cx32 hemichannels are important to detect locally-produced CO_2_. We speculate that a metabolically active group of cells (such as hepatocytes, which abundantly express Cx32^25–27^) might produce very high localized concentrations of CO_2_ that would be sufficient to open Cx32 hemichannels.

**Fig. 10:**
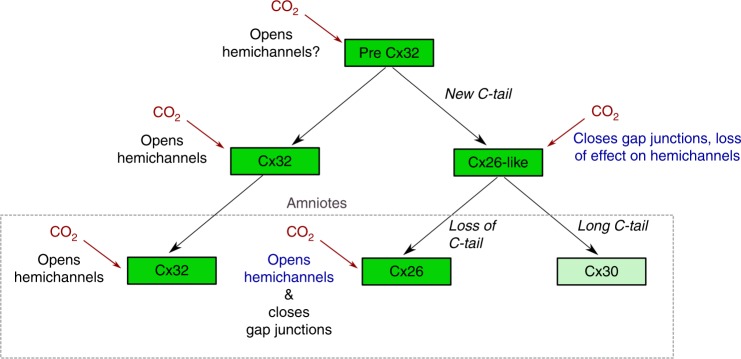
Inferred evolution of CO_2_-dependent functionality in the Cx32 and Cx26-like clades. The common ancestor of the Cx32 and Cx26-like genes (Pre Cx32) most likely had the carbamylation motif (CM). We postulate that this was originally used to regulate the opening of hemichannels; the CM and this functionality has been preserved in Cx32 to the present day. The emergence of the Cx26-like gene was accompanied by a de novo function for the CM — gain of CO_2_-dependent gap junction closure, but at the cost of losing CO_2_-dependent hemichannel opening. In the pre-amniote world, the functions of opening hemichannels and closing of gap junctions were subserved by different gene products. With the evolution of amniotes, the Cx26-like gene was duplicated to give Cx26 and Cx30. Cx30 gained a long C-terminal tail and in many cases lost the carbamylation motif. Cx26 in amniotes lost the C-terminal tail and regained the ability of CO_2_ to open the hemichannel. (Green box indicates near-universal presence of carbamylation motif, light green box presence of carbamylation motif in some species but not others)

We hypothesize (Fig. 10) that the connexin ancestor of the Cx32 and Cx26 clades would have possessed the carbamylation motif and that most likely this motif served to permit CO_2_ opening of the ancestral hemichannel at high levels of PCO_2_ (70 mmHg). When the two clades split, the Cx26-like clade gained a new functionality — ability of CO_2_ to close the gap junction at more modest levels of PCO_2_ (55 mmHg) but simultaneously lost the old functionality — the ability of CO_2_ to open the hemichannels. During the evolution of amniotes, when the Cx26-like gene duplicated to give Cx26 and Cx30, a further evolutionary innovation occurred — loss of the C-terminal tail from the amniote subclade of Cx26. This permitted the opening of Cx26 hemichannels at modest levels of PCO_2_, at a sensitivity range that was appropriate for systemic CO_2_ sensing, and retained the ability of CO_2_ to close the gap junction.

It is striking that Cx26 hemichannels with the structural features that permit opening by CO_2_ have so far only been found in amniotes (Fig. 1, Supplementary Table 2). Equally notable, is that the Cx30 of non-mammalian amniotes lacks the carbamylation motif. Thus, the universal CO_2_ sensor in amniotes is the hemichannel of Cx26 rather than that of Cx30. The key additional step to evolve CO_2_-sensitive Cx26 hemichannels in amniotes was to truncate the extended C-terminal tail. This allowed the repurposing of the carbamylation motif from just closing the Cx26 gap junction (a function present in Cx26 of amphibia and lungfish) to an additional function: opening the hemichannel. In the case of amniote Cx26, less became more: the truncated connexin provided a CO_2_-gated channel capable of releasing ATP into the extracellular space where it could act as an intercellular messenger or neurotransmitter to signal levels of PCO_2_^7,12^. Extant amniotes can trace common ancestry to those that survived the Permo-Triassic catastrophe. This geological event occurred some 250 MYA, involved an increase in global temperatures of some 6 °C, and resulted in extinction of more than 70% of land dwelling forms^28–30^. Given the widespread occurrence of the truncated CO_2_-sensitive Cx26 in amniotes, we hypothesize that this adaptation may have arisen in the ancestors of all extant amniotes that survived this catastrophe.

Extant amniotes can only exchange gases by breathing air — they have no capacity for gas exchange via water. One litre of air contains about 30 times the amount of O_2_ as the same volume of water. Consequently, amniotes can have much lower ventilation rates than water breathing animals. As a result of these lower ventilation rates, air breathing vertebrates accumulate much higher levels of CO_2_ (compared to water-breathers). For example, mammals typically have a PCO_2_ in arterial blood of ~40 mmHg, whereas water breathing fish have a blood PCO_2_ of ~5 mmHg^24^. Amniotes have adapted to the high levels of PCO_2_ by retaining much higher concentrations of ${\mathrm{HCO}}_3^ -$, thus regulating their blood pH to the required physiological levels. Nevertheless, for amniotes, the regulated excretion of CO_2_ and consequent homoeostatic control of acid base balance is a key rate-limiting step critical for life. Amniotes have therefore shifted the primary regulation of breathing from the detection of O_2_ to the detection of CO_2_ and pH^31^. While pH-sensitive mechanisms of central respiratory chemosensitivity are clearly important^10,32–34^, the evolutionary innovation of a CO_2_ sensor (hemichannels of Cx26) capable of releasing ATP in a CO_2_-dependent fashion^6^ is likely to be particularly valuable for amniotes.

Mammals and birds are endothermic and have a high metabolic rate (and hence a high rate of CO_2_ production) to maintain their elevated body temperature. Although reptiles are poikilotherms, they use basking behaviour to elevate their body temperature (and metabolic rates). The arterial PCO_2_ of reptiles is very temperature dependent, but is usually above 20 mmHg, can reach 30–40 mmHg in sun-bathing lizards^35^, and can exceed 40 mmHg in turtles^36,37^. As breathing in turtles responds to variations in PCO_2_ over the range 20–55 mmHg^38^, the CO_2_-sensitivity of Cx26 hemichannels may thus be relevant to the control of breathing in a wide range of amniote species. It is very significant that the EC_50_ of the Cx26 hemichannel is very close to the physiological resting value of PCO_2_ in a variety of species^18^. Our data suggests that there has been strong selective pressure to maintain the CO_2_ sensitivity of Cx26 (both the carbamylation motif and the short C-terminal tail) across the extant amniote lineage.

## Methods

### Phylogenetic and microsyntenic analyses

For the phylogenetic analysis the Cx26, Cx32, and Cx30 orthologous sequences were collected from ENSEMBL or NCBI databases. The *Protopterus annectens* sequence was retrieved from the transcriptome previously published by Biscotti et al.^39^ while the sequence of *Neoceratodus forsteri* was obtained from NCBI (PRJNA317231). *Callorhinchus milii* sequences were collected from http://esharkgenome.imcb.a-star.edu.sg/^40^. Moreover, sequences of genes located in the same genomic regions of those of interest were also added to the phylogenetic analysis. Accession numbers of all sequences used are reported in Supplementary Table 1.

The alignment was performed with MUSCLE (https://www.ebi.ac.uk/Tools/msa/muscle/) using default parameters. The phylogenetic analysis was carried out with MrBayes-3.2^41^. On the basis of the results of microsyntenic analysis the sequence of *Petromyzon marinus* (Cx27.5) was constrained to form a monophyletic clade with those located in the same genomic region. The Jones aa model^42^ was identified by the MrBayes program with a posterior probability of 1.00. The connexin sequence of *Amia calva* was used as the outgroup (accession number GEUG01003334.1); 6,000,000 generations were run and sampling was conducted every 100 generations. Stationarity was defined as the condition where the standard deviation of split frequencies reached 0.0077. The first 15,000 trees were discarded as the burn-in.

The microsyntenic arrangement of the connexin genes here analysed were obtained from ENSEMBL with the exception of *C. milii* obtained from the UCSC Genome Browser (http://genome.cse.ucsc.edu/).

### Generation of connexin expression constructs

The *Cx26* gene sequences from *Latimeria chalumnae* (XM_014493276.1), *Chelonia midas* (XM_007059139.1), *Gekko japonicus* (XM_015429500.1), *Xenopus tropicalis* (CR848317.2), *Lepidosiren paradoxa* (GEHZ01053112.1) and *Cx32* gene sequences from *Danio rerio* (XM_001921588.7) and *Rhincodon typus* (XM_020523441.1) were synthesised by GenScript and subcloned into the pCAG-GS mCherry vector prior to mammalian cell transfection. xtCx26Δ and xtCx26ΔPV were made by creating PCR fragments with the forward primer shown in Table 1 and the reverse primers **XTHLrev2** and **Xen244PVrev**, respectively (Table 1). The presence of the correct Cx26 was confirmed by DNA sequencing (GATC Biotech).

**Table 1:** Primers used in this study

Construct/mutation	Substrate	Forward primer	Reverse primer
xtCx26Δ	*Xenopus* pCAG-Xenopus-mCherry	ATTCGGTACCATGGATTGGGGAACG	**XTHLrev2**, CTCTGGTACCCCTGAATGTTTTTTTGACCTCCTTAGGGAAGC
xtCx26ΔPV	*Xenopus* pCAG-Xenopus-mCherry	ATTCGGTACCATGGATTGGGGAACG	**Xen244PVrev**, GGTATGGTACCCCaactggCTTTTTTGACCTCCTTAGGGAAGC
hCx26 + XenCT	pCAG-humCx26-mCherry	**mchmid**, AGGACGGCGAGTTCATCTAC	**hum-Xen**, agaatgcctgagtgaatgtttTTTTGACTTCCCAGAACAATATC
insert PCR fragment	pCAG-Xenopus-mCherry	**XenTail**, tattgttctgggaagtcaaaaAAACATTCACTCAGGCATTCTAACC	**mch207**, GGTGCTTCACGTAGGCCTTGGAGCCGTACATGAAC
P228G/P229G	human Cx32	ccgagcccagcgccgctccaatGGCGGttcccgcaaggg	cccttgcgggaaccgccattggagcggcgctgggctcgg
P242G	human Cx32	ttcggccaccgcctctcaGGtgaatacaagcag	ctgcttgtattcacctgagaggcggtggccgaa
P268G	human Cx32	catactgcgccgcagcGGtggcaccgggg	ccccggtgccaccgctgcggcgcagtatg
G222P	*Lepidosiren paradoxa* Cx26	ataaaggcatgtctccgtcatCCTaaacaggaaaagtactcgagc	gctcgagtacttttcctgtttaggatgacggagacatgcctttat
G238P	*Lepidosiren paradoxa* Cx26	gctcccagtcaagtttaatgtctaaaCCcaaagagcatcagca	tgctgatgctctttgggtttagacattaaacttgactgggagc

Primer names mentioned in the Methods section are shown in bold. All primers shown in the 5' to 3' direction.

The ‘*Xenopus* Tail’ was added to human Cx26 (to create hCx26 + *Xen*CT of Fig. 5) by Gibson Assembly with the following fragments: the vector fragment was created by PCR from pCAG-humCx26-mCherry^18^ using **hum-Xen** reverse and **mchmid** forward primers (Table 1); the insert PCR fragment was created from pCAG-*Xenopus*-mCherry using **XenTail** forward and **mch207** reverse primers (Table 1).

Mutations to change prolines to glycines in the human Cx32 tail sequence were introduced stepwise by the Quikchange protocol (Agilent) using the primers shown in Table 1 for P228G/P229G, P242G, and P268G. Mutations to change glycines to prolines in the *Lepidosiren paradoxa* Cx26 tail sequence were introduced stepwise using the primers shown in Table 1 for G222P and G238P.

### HeLa cell culture

HeLa DH cells were grown in Dulbecco’s Modified Eagle Medium (DMEM), or HeLa Ohio cells were grown in Eagle’s Minimum Essential Medium, supplemented with 10% fetal bovine serum, 50 μg/mL penicillin/streptomycin and 3 mM CaCl_2_. HeLa DH cells were used for patch clamp studies on all Cx26 variants and for the dye loading of all Cx26 variants except *Latimeria* and hCx26xenCT. For dye loading experiments, cells were plated onto coverslips at a density of 5 × 10^4^ cells per well, and transiently transfected with the Cx26/Cx32 expression constructs following the GeneJuice Transfection Reagent protocol.

### Recording solutions

*Control (35* *mmHg PCO*_*2*_*)*: 124 mM NaCl, 26 mM NaHCO_3_, 1.25 mM NaH_2_PO_4_, 3 mM KCl, 10 mM D-glucose, 1 mM MgSO_4_, 2 mM CaCl_2_. This was bubbled with 95%O_2_/5% CO_2_ and had a final pH of ~7.4.

*Hypercapnic (55* *mmHg PCO*_*2*_*)*: 100 mM NaCl, 50 mM NaHCO_3_, 1.25 mM NaH_2_PO_4_, 3 mM KCl, 10 mM D-glucose, 1 mM MgSO_4_, 2 mM CaCl_2_. This was bubbled with sufficient CO_2_ (~9%, balance O_2_) to give a final pH of ~7.4.

*Hypercapnic (70* *mmHg PCO*_*2*_*)*: 73 mM NaCl, 80 mM NaHCO_3_, 1.25 mM NaH_2_PO_4_, 3 mM KCl, 10 mM D-glucose, 1 mM MgSO_4_, 2 mM CaCl_2_. This was bubbled with sufficient CO_2_ (approximately 12%, balance O_2_) to give a final pH of ~7.4.

*Zero Ca*^*2+*^: 124 mM NaCl, 26 mM NaHCO_3_, 1.25 mM NaH_2_PO_4_, 3 mM KCl, 10 mM D-glucose, 1 mM MgSO_4_, 2 mM MgCl_2_, 1 mM EGTA. This was bubbled with 95%O_2_/5% CO_2_ and had a final pH of ~7.4.

### Dye loading experiments

We used a dye loading protocol that has been developed and extensively described in our prior work^6,12,20,21^. HeLa cells expressing Cx26 for 48–72 h from each of the species tested were initially washed with control solution. They were then exposed to either control or hypercapnic solution containing 200 μM 5(6)-carboxyfluorescein (CBF) for 10 min. Subsequently, cells were returned to control solution with 200 μM CBF for 5 min, before being washed in control solution without CBF for 30 min to remove excess extracellular dye. A replacement coverslip of HeLa cells was used for each condition. For each coverslip, mCherry staining was imaged to verify Cx26 expression. The experiments were replicated independently (independent transfections) at least five times to give *n* = 5 for each species.

### Fluorescence imaging and data analysis

Following dye loading, HeLa cells were imaged by epifluorescence (Scientifica Slice Scope, Cairn Research OptoLED illumination, 60x water Olympus immersion objective, NA 1.0, Hamamatsu ImagEM EM-CCD camera, Metafluor software). ImageJ (Wayne Rasband, National Institutes of Health, USA) was used to measure the extent of dye loading by drawing a region of interest (ROI) around each cell, and subsequently, the mean pixel intensity of the ROI was determined. The mean pixel intensity of a representative background ROI for each image was subtracted from each cell measurement from the same image. At least 40 cells were measured for each condition per experiment, and at least five repetitions of independently transfected HeLa cells were completed. The mean pixel intensities were plotted as cumulative probability distributions and these graphs show every data point measured.

### Patch clamp recordings

Cover slips containing non-confluent HeLa DH cells (preferred over the HeLa Ohio cells for their more rounded morphology) were placed into a chamber and superfused with control saline. An MCI Cleverscope, Photometrics Prime camera and Cairn Instruments OptoLED illumination, and an Olympus 60x water immersion (NA 1.0) objective were used to visualize the cells under brightfield DIC, and mCherry expression (470 nm). Micromanager software was used to control the illumination and camera settings and to save images for offline analysis via ImageJ.

Standard patch clamp techniques were used to make whole-cell patch clamp recordings from HeLa cells that expressed Cx26 as assessed by mCherry fluorescence. The intracellular fluid in the patch pipette contained: K-gluconate 130 mM, KCl 10 mM, EGTA 10 mM, CaCl_2_ 2 mM, HEPES 10 mM, pH adjusted to 7.3 with KOH and was adjusted with pure water to a final osmolarity of 295 mOsm. All whole-cell recordings were performed at a holding potential of −50 mV with steps to −40 mV, lasting 2.5 s and delivered every 5 s, to assess whole-cell conductance.

Imaging of fluorescent tracer movement through gap junctions was achieved by using 2-Deoxy-2-[(7-nitro-2,1,3-benzoxadiazol-4-yl)amino]-D-glucose, NBDG, which was included at 200 µM in the patch recording fluid which was either the same as above or had lowered EGTA concentration (5 mM). Following break-through of the patch pipette to establish the whole-cell mode, images were collected every 10 s. The time required for dye transfer was calculated to be when the acceptor cell reached 10% of the fluorescence of the donor cell.

### Statistical analysis and reproducibility

Data has been plotted as either cumulative probabilities (showing every data point) or box and whisker plots where the box is interquartile rage, bar is median, and whisker extends to most extreme data point that is no more than 1.5 times the interquartile range. All individual data points are superimposed on the plots.

For the patch clamp experiments, an individual replicate is a recording from a single cell. For the gap junction permeation studies, dye transfer between a pair of cells is regarded as a single replicate. For the dye loading studies, single replicate is the analysis of CO_2_ sensitivity from cells resulting from an independent transfection. In this case to avoid pseudoreplication, statistical analysis was performed on the median values arising from each of the independent replicates.

Statistical analysis was performed with the R language. All analysis was performed using the Kruskal–Wallis ANOVA for multiple comparisons and the Mann Whitney *U* test for pairwise comparisons.

### Reporting summary

Further information on research design is available in the Nature Research Reporting Summary linked to this article.

## Supplementary information

Supplementary Information

Description of Additional Supplementary Files

Supplementary Data 1

Reporting Summary

## Data Availability

All data generated or analysed during this study are included in this published article (and its supplementary information files) or are available from the authors upon reasonable request.
