# Supplementary material for: Structural determinants of CO2-sensitivity in the β connexin family suggested by evolutionary analysis
Source: Commun Biol. 2019 Sep 4;2:331. doi: 10.1038/s42003-019-0576-2 (PMC6726660; doi:10.1038/s42003-019-0576-2)
Supplement: Supplementary file 1 — Supplementary Information [file 42003_2019_576_MOESM1_ESM.pdf]

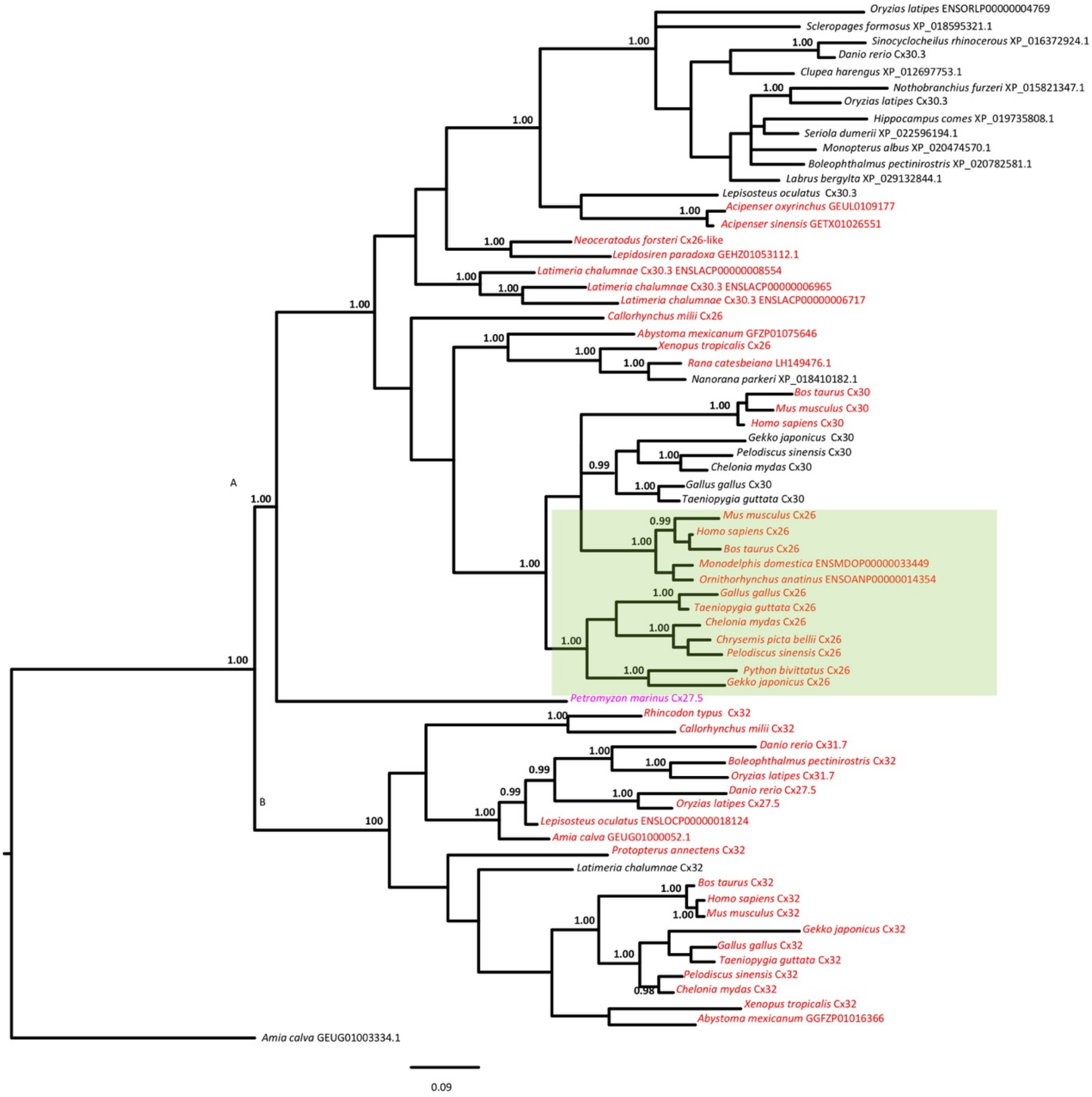

Supplementary Figure 1 Molecular phylogenetic tree of Cx26, Cx30 and Cx32 genes. This analysis includes all of the sequences in Supplementary Tables 1 and 2. Bayesian Inference on amino acid sequences belonging to the main vertebrate lineages. Numbers close to nodes indicate posterior probability values (>0.95). *Amia calva* GEUG01003334.1 (Cx31-like) was used as the outgroup. If the species name is red, the sequence possesses the carbamylation motif. The green box indicates Cx26 with a short C-terminal tail. Two main clades are apparent and labelled A and B.

Methods

The alignment was performed with MUSCLE (https://www.ebi.ac.uk/Tools/msa/muscle/) using default parameters. The phylogenetic analysis was carried out with MrBayes-3.2 1. On the basis of the results of microsyntenic analysis the sequence of *Petromyzon marinus* (Cx27.5) was constrained to form a monophyletic clade with those located in the same genomic region. The Jones aa model 2 was identified by the MrBayes program with a posterior probability of 1.00. The connexin sequence of *Amia calva* was used as the outgroup (accession number GEUG01003334.1); 5,000,000 generations were run and sampling was conducted every 100 generations. Stationarity was defined as the condition

where the standard deviation of split frequencies reached 0.0057. The first 12,500 trees were discarded as the burn-in.

1. Huelsenbeck, J. P., Ronquist, F., Nielsen, R. & Bollback, J. P. Bayesian inference of phylogeny and its impact on evolutionary biology. Science 294, 2310-2314, doi:10.1126/science.1065889 (2001).

2. Jones, D. T., Taylor, W. R. & Thornton, J. M. The rapid generation of mutation data matrices from protein sequences. Comput Appl Biosci 8, 275-282 (1992).

Supplementary Tables

Class	Order	Species	Gene	Accession number
Mammalia	Primates	*Homo sapiens*	Cx32	ENSP00000363134
			Cx26	ENSP00000495035
			Cx30	ENSP00000348521
	Rodentia	*Mus musculus*	Cx32	ENSMUSP00000113516
			Cx26	ENSMUSP00000054343
			Cx30	ENSMUSP00000035630
	Artiodactyla	*Bos taurus*	Cx32	ENSBTAP00000027331
			Cx26	ENSBTAP00000023167
			Cx30	ENSBTAP00000052852
Aves	Galliformes	*Gallus gallus*	Cx32	ENSGALP00000050756
			Cx26	ENSGALP00000035659
			Cx30	ENSGALP00000027628
	Passeriformes	*Taeniopygia guttata*	Cx32	ENSTGUP00000007332
			Cx26	ENSTGUP00000011656
			Cx30	ENSTGUP00000011657
Sauropsida	Testudines	*Pelodiscus sinensis*	Cx32	ENSPSIP00000000180
			Cx26	ENSPSIP00000001776
			Cx30	ENSPSIP00000001778
		Chelonia mydas	Cx32	XP_007056181.1
			Cx26	XP_007059201.1
			Cx30	XP_007059188.1
	Squamata	*Gekko japonicus*	Cx32	XP_015264464.1
			Cx26	XP_015284986.1
			Cx30	XP_015284969.1
Amphibia	Anura	*Xenopus tropicalis*	Cx32	ENSXETP00000055497
			Cx26	ENSXETP00000055228

		Abystoma mexicanum	Cx32	GGFZP01016366.1
			Cx26	GFZP01075646.1
Sarcopterygii	Coelacanthiformes	*Latimeria chalumnae*	Cx32	ENSLACP00000004817
			Cx30.3	ENSLACP00000006965
			Cx30.3	ENSLACP00000006717
			Cx30.3	ENSLACP00000008554
		Protopterus annectens	Cx32	MN231038
		Neoceratodus forsteri	Cx26	MN231039
		Lepidosiren paradoxa	Cx26	GEHZ01053112.1
Actinopterygii	Lepisosteiformes	*Lepisosteus oculatus*	Cx30.3	ENSLOCP00000021909
				ENSLOCP00000018124
	Acipenseriformes	*Acipenser oxyrinchus*	Cx30.3	GEUL01091774.1
		Acipenser sinensis	Cx32	GETX01026551.1
	Cypriniformes	*Danio rerio*	Cx30.3	ENSDARP00000062668
			Cx27.5	ENSDARP00000051538
			Cx31.7	ENSDARP00000124413
	Beloniformes	*Oryzias latipes*	Cx30.3	ENSORLP00000016225
			Cx30.3	ENSORLP00000004769
			Cx27.5	ENSORLP00000011896
			Cx31.7	ENSORLP00000008192
	Amiiformes	*Amia calva*	Cx32	GEUG01000052.1
	Perciformes	*Boleophthalmus pectinirostris*	Cx26	XP_020782581.1
			Cx32	XP_020784229.1
Chondrichthyes	Chimaeriformes	*Callorhinchus milii*	Cx32	SINCAMP00000026088
			Cx26	SINCAMP00000001808
	Orectolobiformes	*Rhincodon typus*	Cx32	XP_020379030.1
Petromyzontiformes	Petromyzontiformes	*Petromyzon marinus*	Cx27.5	ENSPMAP00000009847

Supplementary Table 1. Accession numbers of sequences used in the phylogenetic analysis of Figures 1 and 2.

Species	Accession	Carbamylation motif	C-terminal tail
Sinocyclocheilus rhinocerous	XP_016372924.1	No	Long
Clupea harengus	XP_012697753.1	No	Long
Monopterus albus	XP_020474570.1	No	Long
Labrus bergylta	XP_020487680.1	No	Long
Hippocampus comes	XP_019735808.1	No	Long
Scleropages formosus	XP_018595321.1	No	Long
Seriola dumerili	XP_022596194.1	No	Long
Nothobranchius furzeri	XP_015821347.1	No	Long
Rana catesbeiana	LH149476.1	Yes	Long
Nanorana parkeri	XP_018410182.1	No	Long
Chrysemis picta bellii	XP_005312279.1	Yes	Short
Python bivittatus	XP_007427604.1	Yes	Short
Monodelphis domestica	ENSMODP00000033449	Yes	Short
Ornithorhynchus anatinus	ENSOANP00000014354	Yes	Short

Supplementary Table 2. Analysis of Cx26 from additional species, not shown in Figure 1 of main paper, to indicate presence of carbamylation motif and length of C-terminal tail. Amniotes are shaded in green.
