# Supplementary material for: Structural determinants of CO2-sensitivity in the β connexin family suggested by evolutionary analysis
Source: Commun Biol. 2019 Sep 4;2:331. doi: 10.1038/s42003-019-0576-2 (PMC6726660; doi:10.1038/s42003-019-0576-2)
Supplement: Supplementary file 2 — Description of Additional Supplementary Files [file 42003_2019_576_MOESM2_ESM.docx]

**Description of Additional Supplementary Files**

**File Name**: **Supplementary Data 1**

**Description**:   Data for figures in text
