# Supplementary material for: Structural determinants of CO2-sensitivity in the β connexin family suggested by evolutionary analysis
Source: Commun Biol. 2019 Sep 4;2:331. doi: 10.1038/s42003-019-0576-2 (PMC6726660; doi:10.1038/s42003-019-0576-2)
Supplement: Supplementary file 4 — Reporting Summary [file 42003_2019_576_MOESM4_ESM.pdf]

nature research

Corresponding author(s): Nicholas Dale

Life Sciences Reporting Summary

Nature Research wishes to improve the reproducibility of the work that we publish. This form is intended for publication with all accepted life science papers and provides structure for consistency and transparency in reporting. Every life science submission will use this form; some list items might not apply to an individual manuscript, but all fields must be completed for clarity.

For further information on the points included in this form, see Reporting Life Sciences Research. For further information on Nature Research policies, including our data availability policy, see Authors & Referees and the Editorial Policy Checklist.

Please do not complete any field with "not applicable" or n/a. Refer to the help text for what text to use if an item is not relevant to your study.
For final submission: please carefully check your responses for accuracy; you will not be able to make changes later.

Experimental design

1. Sample size

Describe how sample size was determined.

Based on sample sizes from previous work that showed clear effects of CO_2 on Cx26 hemichannels

2. Data exclusions

Describe any data exclusions.

No exclusions

3. Replication

Describe the measures taken to verify the reproducibility of the experimental findings.

Each dye-loading experiment was independently replicated at least 5 times (i.e. at least 5 independent transfections of relevant gene). Results from dye loading were independently checked by whole cell patch clamp -a completely different methodology. Patch clamp experiments were performed on multiple independently transfected batches of cells.

4. Randomization

Describe how samples/organisms/participants were allocated into experimental groups.

n/a

5. Blinding

Describe whether the investigators were blinded to group allocation during data collection and/or analysis.

Blinding is not customary in electrophysiological experiments and would be hard as cells were selected for analysis by their expression of the mCherry reporter. Blinding not performed on dye loading experiments for similar reason. The two sets of experiments were performed independently by different investigators and exactly correspond indicating that there is no bias.

Note: all in vivo studies must report how sample size was determined and whether blinding and randomization were used.

6. Statistical parameters

For all figures and tables that use statistical methods, confirm that the following items are present in relevant figure legends (or in the Methods section if additional space is needed).

n/a	Confirmed	
☐	☒	The exact sample size (*n*) for each experimental group/condition, given as a discrete number and unit of measurement (animals, litters, cultures, etc.)
☐	☒	A description of how samples were collected, noting whether measurements were taken from distinct samples or whether the same sample was measured repeatedly
☐	☒	A statement indicating how many times each experiment was replicated
☐	☒	The statistical test(s) used and whether they are one- or two-sided<br>*Only common tests should be described solely by name; describe more complex techniques in the Methods section.*
☒	☐	A description of any assumptions or corrections, such as an adjustment for multiple comparisons
☐	☒	Test values indicating whether an effect is present<br>*Provide confidence intervals or give results of significance tests (e.g. P values) as exact values whenever appropriate and with effect sizes noted.*
☐	☒	A clear description of statistics including central tendency (e.g. median, mean) and variation (e.g. standard deviation, interquartile range)
☐	☒	Clearly defined error bars in all relevant figure captions (with explicit mention of central tendency and variation)

See the web collection on statistics for biologists for further resources and guidance.

Software

Policy information about availability of computer code

7. Software

Describe the software used to analyze the data in this study.	R language

For manuscripts utilizing custom algorithms or software that are central to the paper but not yet described in the published literature, software must be made available to editors and reviewers upon request. We strongly encourage code deposition in a community repository (e.g. GitHub). *Nature Methods* guidance for providing algorithms and software for publication provides further information on this topic.

Materials and reagents

Policy information about availability of materials

8. Materials availability

Indicate whether there are restrictions on availability of unique materials or if these materials are only available for distribution by a third party.	All materials readily available from commercial sources (given in Methods).

9. Antibodies

Describe the antibodies used and how they were validated for use in the system under study (i.e. assay and species).	No antibodies were used.

10. Eukaryotic cell lines

a. State the source of each eukaryotic cell line used.	HeLa DH and HeLa Ohio
b. Describe the method of cell line authentication used.	None of the cell lines have been authenticated but were obtained from ATCC for this study.
c. Report whether the cell lines were tested for mycoplasma contamination.	Cells tested and negative for mycoplasma contamination
d. If any of the cell lines used are listed in the database of commonly misidentified cell lines maintained by ICLAC, provide a scientific rationale for their use.	No commonly misidentified cell lines were used.

November 2017

Animals and human research participants

Policy information about studies involving animals; when reporting animal research, follow the ARRIVE guidelines

11. Description of research animals

Provide all relevant details on animals and/or animal-derived materials used in the study.	n/a

Policy information about studies involving human research participants

12. Description of human research participants

Describe the covariate-relevant population characteristics of the human research participants.	n/a
